# GDF15 Protects Insulin-Producing Beta Cells against Pro-Inflammatory Cytokines and Metabolic Stress via Increased Deamination of Intracellular Adenosine

**DOI:** 10.3390/ijms25020801

**Published:** 2024-01-08

**Authors:** Anongnad Ngamjariyawat, Jing Cen, Xuan Wang, Nils Welsh

**Affiliations:** 1 Science for Life Laboratory, Department of Medical Cell Biology, Uppsala University, Box 571, SE-751 23 Uppsala, Sweden; anongnad.ngamjariyawat@mcb.uu.se (A.N.); jing.cen@mcb.uu.se (J.C.); xuan.wang@mcb.uu.se (X.W.); 2 Division of Anatomy, Faculty of Medicine, Thammasat University, Pathumthani 12120, Thailand

**Keywords:** apoptosis, beta cell, GDF15, imatinib, insulin production, mitochondrial respiration, adenosine, adenosine deaminase

## Abstract

It has been proposed that antidiabetic drugs, such as metformin and imatinib, at least in part, promote improved glucose tolerance in type 2 diabetic patients via increased production of the inflammatory cytokine GDF15. This is supported by studies, performed in rodent cell lines and mouse models, in which the addition or production of GDF15 improved beta-cell function and survival. The aim of the present study was to determine whether human beta cells produce GDF15 in response to antidiabetic drugs and, if so, to further elucidate the mechanisms by which GDF15 modulates the function and survival of such cells. The effects and expression of GDF15 were analyzed in human insulin-producing EndoC-betaH1 cells and human islets. We observed that alpha and beta cells exhibit considerable heterogeneity in GDF15 immuno-positivity. The predominant form of GDF15 present in islet and EndoC-betaH1 cells was pro-GDF15. Imatinib, but not metformin, increased pro-GDF15 levels in EndoC-betaH1 cells. Under basal conditions, exogenous GDF15 increased human islet oxygen consumption rates. In EndoC-betaH1 cells and human islets, exogenous GDF15 partially ameliorated cytokine- or palmitate + high-glucose-induced loss of function and viability. GDF15-induced cell survival was paralleled by increased inosine levels, suggesting a more efficient disposal of intracellular adenosine. Knockdown of adenosine deaminase, the enzyme that converts adenosine to inosine, resulted in lowered inosine levels and loss of protection against cytokine- or palmitate + high-glucose-induced cell death. It is concluded that imatinib-induced GDF15 production may protect human beta cells partially against inflammatory and metabolic stress. Furthermore, it is possible that the GDF15-mediated activation of adenosine deaminase and the increased disposal of intracellular adenosine participate in protection against beta-cell death.

## 1. Introduction

Growth differentiation factor 15 (GDF15) is a cytokine that belongs to the transforming growth factor-β family. In young and healthy individuals, GDF15 levels are usually low, but can increase dramatically in response to injury, inflammation, pathogens, mitochondrial stress, and other pathological conditions [1,2,3]. This makes GDF15 a prognostic factor for diseases like cardiac disease, lung disease, cancer, neurodegenerative disease, and metabolic syndrome [4,5,6,7,8]. GDF15 is not only a biomarker; it can also exert direct effects in certain regions in the brain via an activation of the glial-cell-derived neurotrophic factor family receptor alpha-like (GFRAL), which, together with its coreceptor rearranged during transfection (RET), signals to reduce food intake and improve energy balance [9,10,11]. GDF15 also affects other parts of the body, but in these cases, it is not clear by which mechanisms/receptors it acts. GDF15’s effects are considered pleiotropic, as both beneficial and adverse effects have been observed. It is assumed that GDF15-mediated outcomes vary considerably depending on the circumstances, such as GDF15 levels, GDF15 maturation, duration of GDF15 production, interaction with other cytokines/hormones, cell/tissue type, exercise, and age [12]. 

In type 1 and type 2 diabetes (T1D and T2D), the function and survival of insulin-producing beta cells are reduced. Both inflammatory and metabolic stress have been shown to reduce insulin production and beta-cell viability, but the exact mechanisms by which beta-cell sensitivity to different stress signals is controlled are not fully understood. Fortunately, a better understanding of how antidiabetic drugs modulate beta-cell signaling can provide valuable clues to achieve improved beta-cell survival. One such antidiabetic drug is metformin, which is known to promote increased GDF15 production in individuals with T2D [13,14]. This occurs via metformin-induced activation of the AMP-dependent kinase (AMPK), which, in turn, stimulates GDF15 production [15]. As GDF15 promotes reduced food intake and improved glucose homeostasis, it has been proposed that the antidiabetic actions of metformin are, at least in part, mediated via increased GDF15 [16,17]. This notion is supported by recent observations reporting that GDF15 improves mouse beta-cell function in in vitro and in vivo models [18,19,20,21,22]. A second drug of interest is the tyrosine kinase inhibitor imatinib, which has been reported to promote antidiabetic effects in both T1D and T2D [23,24]. Imatinib is known to improve beta-cell function and viability [25], and, similar to metformin, it activates AMPK [26]. This opens the possibility that metformin and imatinib both protect beta cells against inflammatory or metabolic stress by enhancing AMPK-induced GDF15 production. The aim of the present investigation was, therefore, to study whether beta-cell GDF15 production is stimulated by metformin or imatinib and, if so, to further elucidate the mechanisms by which GDF15 modulates beta-cell function and survival. We report here that imatinib enhances GDF15 production in human beta cells and that GDF15 protects against stress-induced beta-cell death by increasing the disposal of intracellular adenosine.

## 2. Results

### 2.1. GDF15 Protein Expression in Human Islet and EndoC-betaH1 Cells

**Figure 1:**
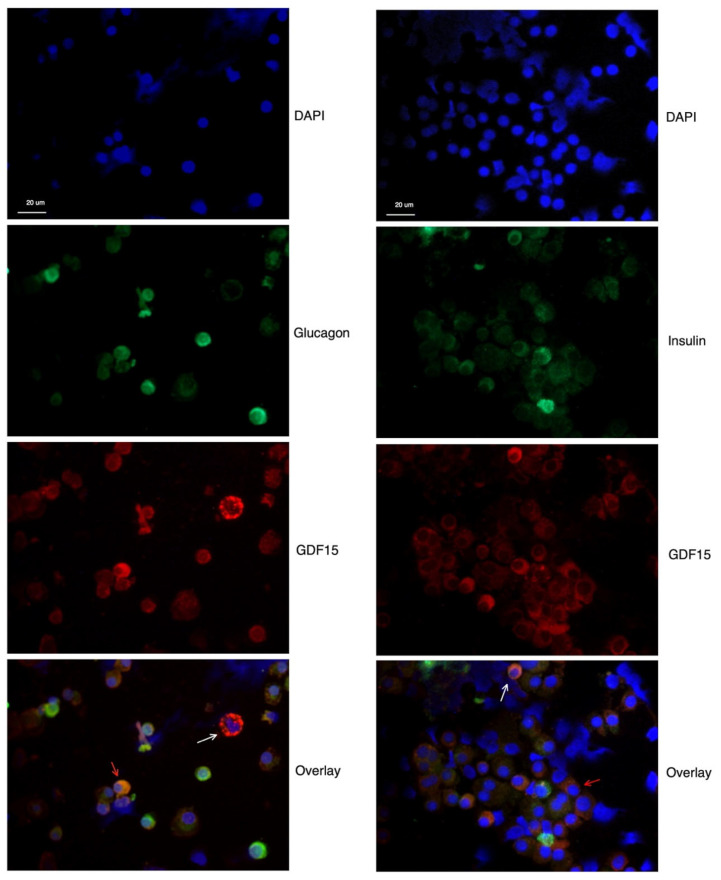
GDF15 immunostaining in glucagon- or insulin-positive human islet cells. Dispersed human islet cells were stained with rabbit anti-GDF15 antibody (red), mouse anti-glucagon (green), guinea pig anti-insulin (green), and DAPI (blue). The white arrows point to cells that were GDF15 positive and glucagon or insulin negative, whereas the orange arrows point to the colocalization of GDF15 with glucagon or insulin in the cytoplasm of the cells. Scale bar, 20 μm.

To assess the expression patterns of the GDF15 protein in human islet cells, dispersed human pancreatic islets were immunostained for GDF15, insulin, and glucagon. As depicted in Figure 1, variable GDF15 (red) immunostaining was observed in the cytoplasm of human islet cells, with some cells exhibiting strong GDF15 signals, and others were more or less devoid of GDF15 immunoreactivity (Figure 1). Strong GDF15 immunostaining colocalized in some cells with insulin (green) or glucagon immunoreactivities (green) (Figure 1, orange arrows). Some cells were GDF15 positive but insulin or glucagon negative (Figure 1, white arrows). Thus, both beta and alpha cells express GDF15, but there exists considerable heterogeneity in the GDF15 expression levels within the two cell types in human islets.

**Figure 2:**
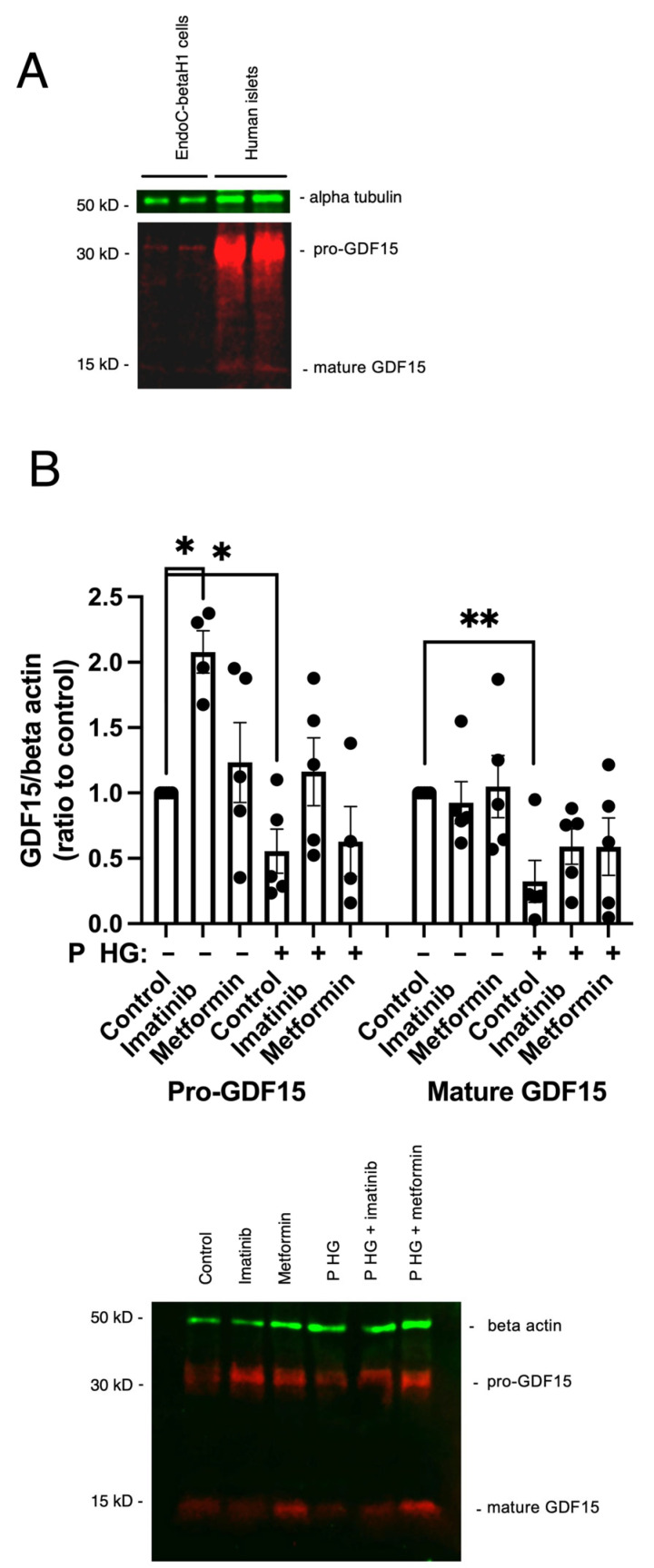
Imatinib increases GDF15 protein levels in EndoC-betaH1 cells. (**A**) Immunoblot analysis of GDF15 and alpha-tubulin in total cell extracts from human islets and EndoC-betaH1 cells. GDF15 migrates as an upper 35 kD band (pro-GDF15) and as a lower 13 kD band (mature GDF15). Human islets appear to express more GDF15 upper band than EndoC-betaH1 cells. (**B**) EndoC-betaH1 cells were precultured with 10 μM imatinib or 0.5 mM metformin for 6 h prior and then treated without or with 1.5 mM palmitate (P) + 22 mM glucose (HG) for 24 h before harvesting. Whole cell lysates were analyzed by immunoblotting with GDF15 and beta-actin antibodies. The ratios of GDF15 (red) to beta-actin (green), for both the upper and the lower bands, were calculated and normalized to the control condition. The results are the mean ± SEM for 4–5 independent experiments. *p*-Values smaller than 0.05 and 0.01 were denoted with * and **, respectively.

The expression of GDF15 was further confirmed by immunoblotting of human islets and EndoC-betaH1 cells. GDF15 is synthesized first as a pro-GDF15 monomer (~40 kDa), which dimerizes and is subsequently cleaved by a furin protease to the mature GDF15 dimer (~30 kDa) [27]. In immunoblot experiments, we observed strong pro-GDF15 monomer (~35 kDa) and weak mature GDF15 monomer (~13 kDa) immunoreactivities (Figure 2A). Pro-GDF15 was considerably stronger in human islets compared with EndoC-betaH1 cells (Figure 2A). In our previous study, in which RNA sequencing was performed on human islets and EndoC-betaH1 cells [28], the levels of GDF15 mRNA were considerably higher in human islets than in EndoC-betaH1 cells (Supplementary Figure S1). Therefore, compared with EndoC-betaH1 cells, GDF15 is more robustly expressed in human islets at both the mRNA and protein levels. 

GFRAL has been described as a GDF15 receptor in certain neuronal cells, but its expression is known to be absent in many other cell types [9]. Our previous RNA-seq results indicate that the GDF15 receptor GFRAL is not expressed in human islets and EndoC-betaH1 cells (Supplementary Figure S1). Thus, it is possible that the GDF15 receptor in beta cells is not GFRAL. However, other putative GDF15 receptors are the receptors belonging to the TGF-beta receptor family, ALK1-7, which were expressed at various levels in both human islets and EndoC-betaH1 cells (Supplementary Figure S1). 

### 2.2. Imatinib Increases GDF15 Protein Expression in EndoC-betaH1 Cells

Imatinib and metformin are known to have beneficial effects on beta-cell function and viability [26,29,30]. In addition, in a previous study we observed that imatinib increases GDF15 mRNA levels in human islets, as assessed by RNA sequencing (1350 ± 290 vs. 1840 ± 360 FPKM, *n* = 9, *p* < 0.05 using a paired *t*-test) [26]. We therefore presently analyzed whether imatinib or metformin affected GDF15 protein levels in EndoC-betaH1 cells. Cells were pre-incubated for 6 h with 10 μM imatinib or 0.5 mM metformin followed by additional treatment with palmitate + high glucose for 24 h. Immunoblot analysis revealed that imatinib, but not metformin, increased pro-GDF15 under control conditions (Figure 2B). Palmitate + high-glucose treatment reduced both pro-GDF15 and mature GDF15 levels (Figure 2B). In the presence of palmitate + high glucose, imatinib tended to increase pro-GDF15, but this did not reach statistical significance. Mature GDF15 was not affected by imatinib. Thus, imatinib increases pro-GDF15 production both at the mRNA and protein levels, suggesting that the beneficial effects of imatinib, at least in part, may be mediated via increased expression of GDF15.

### 2.3. The Proliferation of EndoC-betaH1 Cells Is Unaffected by GDF15

GDF15 has been reported to maintain beta-cell proliferation in streptozotocin-treated mice [18]. To determine if GDF15 affects EndoC-betaH1 cell proliferation, we quantified EndoC-betaH1 cell numbers during a 0-to-4-day incubation period in the presence of GDF15. We found that neither a low (10 ng/mL) nor a high dose (1000 ng/mL) of GDF15 affected cell numbers as compared with the control cells (Supplementary Figure S2).

### 2.4. ATP-Coupled Oxygen Consumption Is Stimulated by GDF15 under Control Conditions but Not in Cytokine-Treated Human Islets

**Figure 3:**
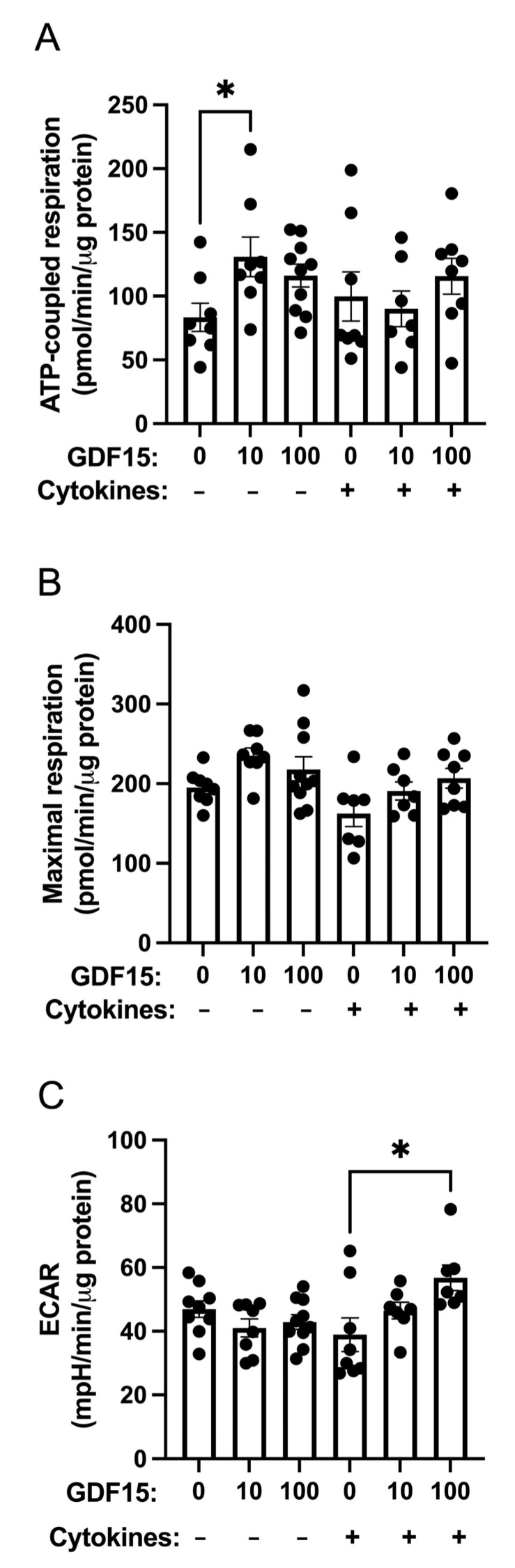
GDF15 increases ATP-coupled mitochondrial oxygen consumption rates under control conditions. Human islets were preincubated without or with GDF15 (10 ng/mL or 100 ng/mL) for 24 h prior to treatment with 20 ng/mL IL-1β + 20 ng/mL IFN-γ (Cytokines) for 48 h followed by oxygen consumption rate (OCR) and extracellular acidification rate (ECAR) determinations using the Seahorse analyzer. (**A**) depicts ATP-coupled respiration. (**B**) represents maximal respiration in the presence of FCCP. (**C**) depicts ECAR. The results are the mean ± SEM from 2 independent observations, each with 3–6 replicates. * denotes *p* < 0.05.

GDF15 has been reported to stimulate the OCR in neuronal cells [31]. We, therefore, analyzed OCRs in human islets under basal conditions and during cytokine (IL-1β + IFN-γ)-induced inflammatory stress. GDF15 treatment increased the ATP-coupled OCR under basal conditions but not in cytokine-treated islet cells (Figure 3). Maximal respiration, in the presence of the uncoupler FCCP, was unaffected by both cytokines and GDF15 (Figure 3B). The extracellular acidification rates (ECARs), which often reflect glycolysis rates, were increased by GDF15 in cytokine-exposed cells (Figure 3C).

### 2.5. GDF15 Increases Insulin Release and Content in EndoC-betaH1 Cells Treated with Cytokines or Palmitate + High Glucose

**Figure 4:**
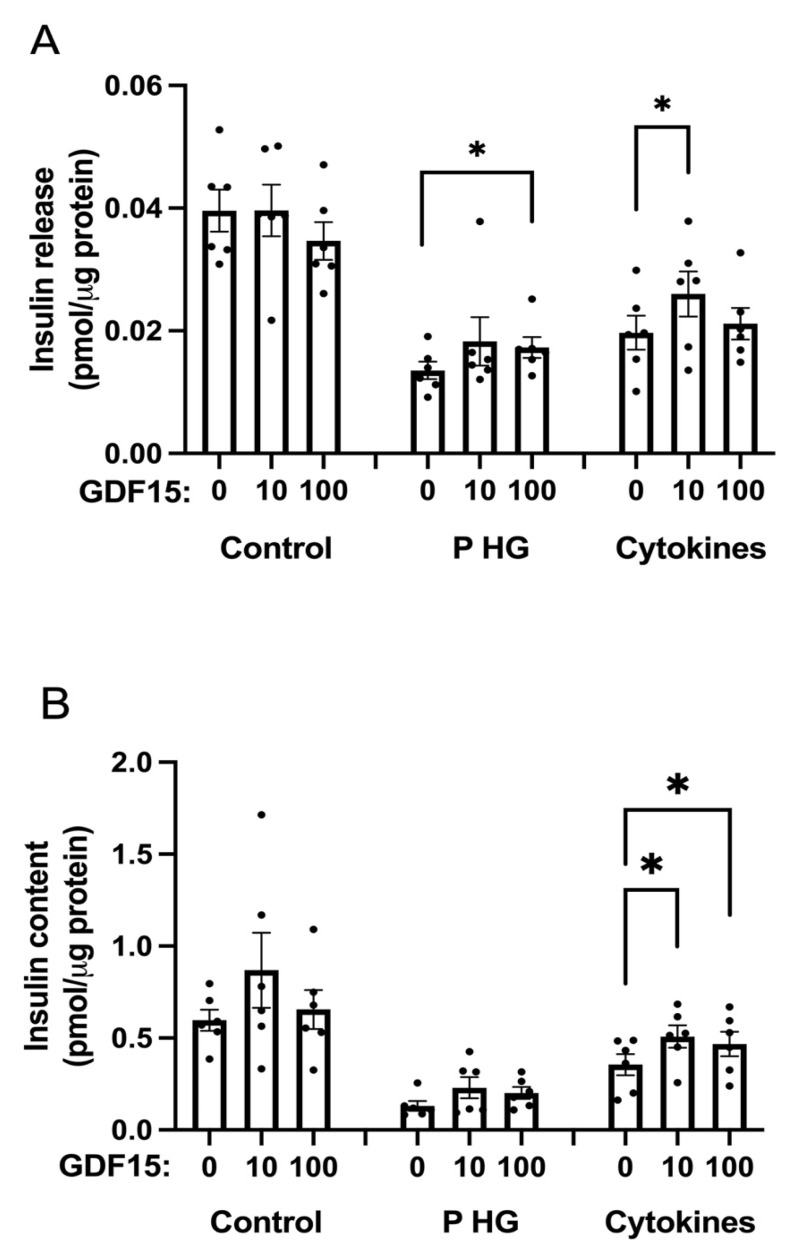
GDF15 increases insulin release and insulin content in EndoC-betaH1 cell exposed to cytokines or palmitate (P) + high glucose (HG). EndoC-betaH1 cells were pretreated without or with GDF15 (10 ng/mL or 100 ng/mL) for 24 h. The incubation was then continued for another 24 with or without cytokines (20 ng/mL IL-1β + 20 ng/mL IFN-γ) or P HG (1.5 mM palmitate + 22 mM glucose). (**A**) Cells were incubated in high-glucose (16.7 mM) KRBH buffer for 1 h after which insulin release (**A**) and insulin content (**B**) levels were analyzed using a Mercodia insulin ELISA assay kit. Protein was quantified using Bradford protein assay. The results were normalized to control condition and are the mean ± SEM from 6 independent experiments. * indicates *p* < 0.05 in comparison to control.

GDF15 has been reported to stimulate insulin release from mouse pancreatic islets and INS-1 cells [19,20]. However, effects of GDF15 on human EndoC-betaH1 cells, exposed to pro-inflammatory cytokines or gluco/lipotoxic conditions, has, to our knowledge, hitherto not been investigated. We therefore pretreated EndoC-betaH1 cells with GDF15 for 24 h and continued the exposure period for another 24 h with or without cytokine (IL-1β + IFN-γ) or palmitate + high-glucose treatments. The release of insulin during a 1 h incubation at 16.7 mM glucose was quantified. Both insulin release and content were markedly reduced when cells were treated with cytokines or palmitate + high glucose (Figure 4A,B). GDF15 did not affect insulin release or insulin content under control conditions (Figure 4A,B). However, the insulin levels secreted from cytokine and palmitate + high-glucose-exposed cells were increased by GDF15. Also, the insulin contents in cytokine-exposed cells were increased by GDF15 (Figure 4B). GDF15 tended to increase insulin contents of palmitate + high-glucose exposed cells, without reaching statistical significance. These results suggest that GDF15 protects human beta cells against inflammatory and metabolic stress-induced beta-cell dysfunction.

### 2.6. GDF15 Partially Protects EndoC-betaH1 and Human Islet Cells from Palmitate + High-Glucose- and Cytokine-Induced Cell Death

**Figure 5:**
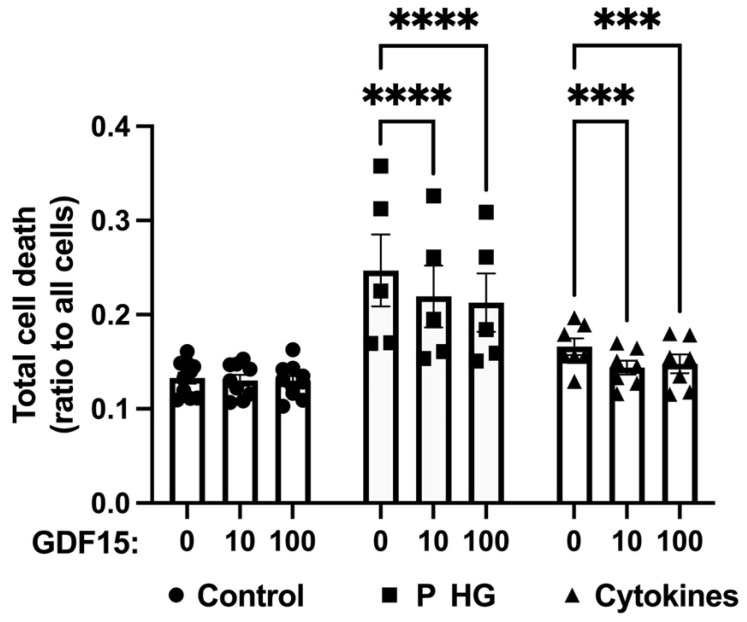
GDF15 partially protects against palmitate (P) + high-glucose (HG)- and cytokine-induced EndoC-betaH1 cell death. EndoC-betaH1 cells were cultured with GDF15 (10 ng/mL or 100 ng/mL) for 24 h followed by additional treatments with cytokines (IL-1β + IFN-γ) or P HG (1.5 mM palmitate + 22 mM glucose) for another 24 h. Cells were stained with Annexin V and PI for flow cytometry analysis of total cell death. The results are presented as the mean ± SEM from 5 to 9 independent observations; *** and **** indicate *p* < 0.001 and *p* < 0.0001, respectively.

GDF15 has been reported to both counteract and promote mouse beta-cell apoptosis [21,32]. We therefore investigated whether GDF15 affects palmitate + high-glucose- and cytokine-mediated human EndoC-betaH1 cell death. EndoC-betaH1 cells were incubated with or without GDF15 for 24 h prior to left treated or untreated with IL-1β + IFN-γ or palmitate + high glucose for an additional 24 h. Flow cytometry analysis of Annexin V (apoptosis) and PI (necrosis) was performed, and total cell death was quantified. We observed that palmitate + high-glucose or cytokine exposure increased the total cell death rates compared with the control cells (Figure 5). GDF15 did not affect EndoC-betaH1 cell survival under control conditions, but it protected partially against cytokine- or palmitate + high-glucose-induced cell death (Figure 5).

**Figure 6:**
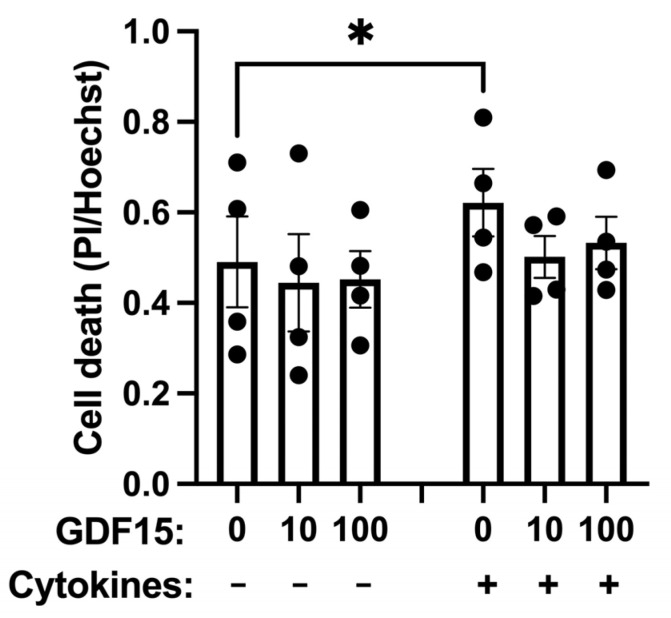
Cytokine-induced human islet cell death is counteracted by GDF15. Human islets were preincubated with 10 ng/mL or 100 ng/mL GDF15 for 24 h. Cells were then treated with cytokines with continued presence of GDF15 for additional 24 h. Dead cells were stained with PI, while all cell nuclei were labeled by Hoechst. The PI/Hoechst ratio was calculated as a relative measure of islet cell death and presented as the mean ± SEM of 4 independent experiments. * Denotes *p* < 0.05 in comparison to the control.

We next addressed the question of whether GDF15 also protects human pancreatic islets from pro-inflammatory cytokines. Therefore, we incubated human islets for 24 h with or without GDF15 prior to cytokine exposure for another 24 h. Cell death was examined by labeling with propidium iodide (PI) and Hoechst. As expected, IL-1β + IFN-ɣ treatment significantly induced cell death in human islets, as indicated by an increase in the amount of PI/Hoechst ratio (Figure 6). In the presence of GDF15, however, IL-1β + IFN-ɣ treatment did not increase human islet cell death. Thus, GDF15 may improve the viability of human beta cells exposed to pro-inflammatory cytokine stress.

### 2.7. GDF15 Stimulates Adenosine Deminase (ADA)-Mediated Conversion of Adenosine to Inosine

**Figure 7:**
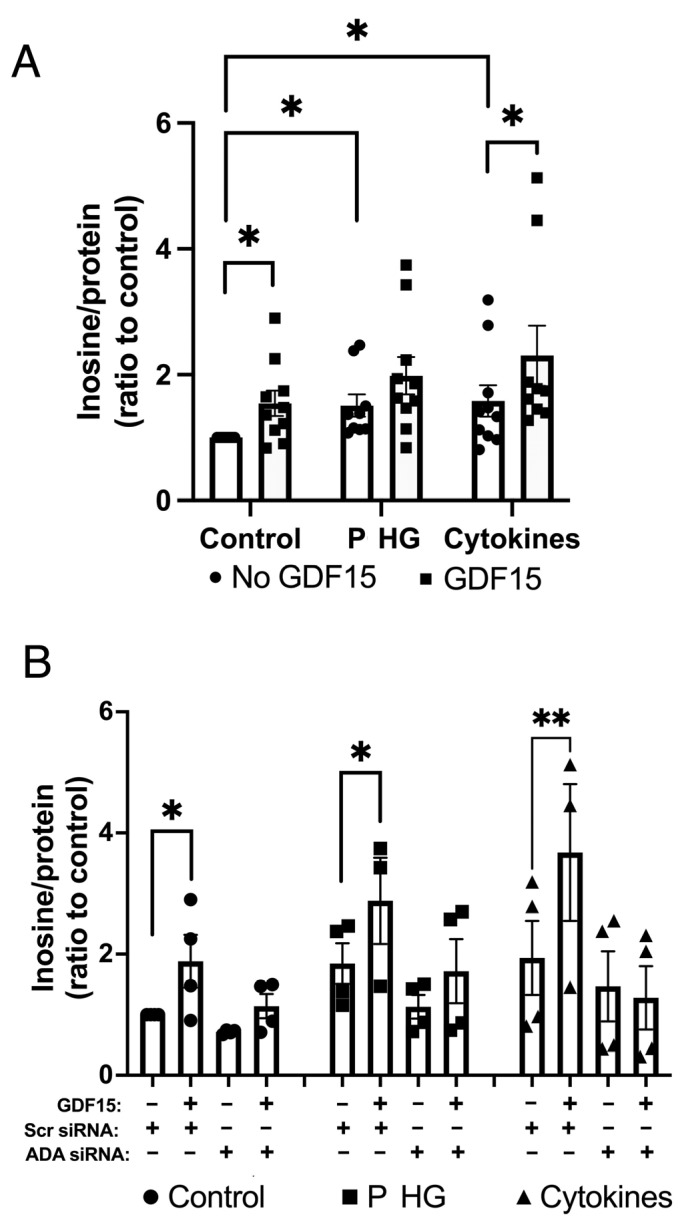
GDF15 promotes ADA-induced adenosine-to-inosine conversion. (**A**) EndoC-betaH1 cells were preincubated with 100 ng/mL GDF15 for 24 h and then exposed to 20 ng/mL IL-1β + 20 ng/mL IFN-γ (cytokines) or 1.5 mM palmitate + 22 mM glucose (P HG) for an additional 24 h. Cells were incubated for 60 min in a KRBH buffer after which inosine levels were quantified using an inosine assay kit and normalized to total protein contents. The results were normalized to the scramble (Scr) control and are the means ± SEM (*n* = 10). (**B**) EndoC-betaH1 cells were lipofected with Scr or ADA siRNA one day prior to the start of the treatments as given in (**A**). Cells were then incubated in KRBH buffer 1 h before supernatants and cells were collected for inosine assay. The results are shown as the means ± SEM of 4 independent experiments; * and ** indicate *p* < 0.05 and *p* < 0.01, respectively.

We recently reported that high intracellular concentrations of adenosine promote beta-cell death via inhibition of the PI3K-Akt-Bad survival pathway [33]. To evaluate the possible involvement of adenosine in GDF15-mediated protection of EndoC-betaH1 cells, we pretreated with GDF15 and then with IL-1β (20 ng/mL) + IFN-γ (20 ng/mL) or palmitate (1.5 mM) + glucose (22 mM) for an additional 24 h after which inosine was determined. Inosine is the deamination product of adenosine and inosine levels can be taken as an indication of adenosine deaminase activity. Stress treatment (cytokines or palmitate + high glucose) promoted increased inosine levels, suggesting adenosine nucleotide dephosphorylation and rising intracellular adenosine levels (Figure 7A), as previously reported [33]. In the GDF15-treated groups, inosine was further increased under control and cytokine conditions. At palmitate + high-glucose conditions, there was a nonsignificant trend to increased inosine in GDF15-treated cells (Figure 7A). This suggests that GDF15 increases the conversion of adenosine to inosine, thereby possibly improving beta-cell survival. To study whether GDF15 stimulates adenosine-to-inosine conversion via increased ADA activity, we lipofected EndoC-betaH1 cells with scramble (Scr) or ADA siRNA before the treatments described for Figure 7A started. Cells were then assayed for inosine levels. We observed that ADA siRNA prevented the GDF15-induced increase in inosine under control, palmitate + high-glucose, and cytokine conditions (Figure 7B). These results suggest that GDF15-induced inosine production, as well as the simultaneous consumption of intracellular adenosine, requires ADA activation.

### 2.8. ADA Downregulation Counteracts GDF15-Induced Protection against EndoC-betaH1 Cell Death

**Figure 8:**
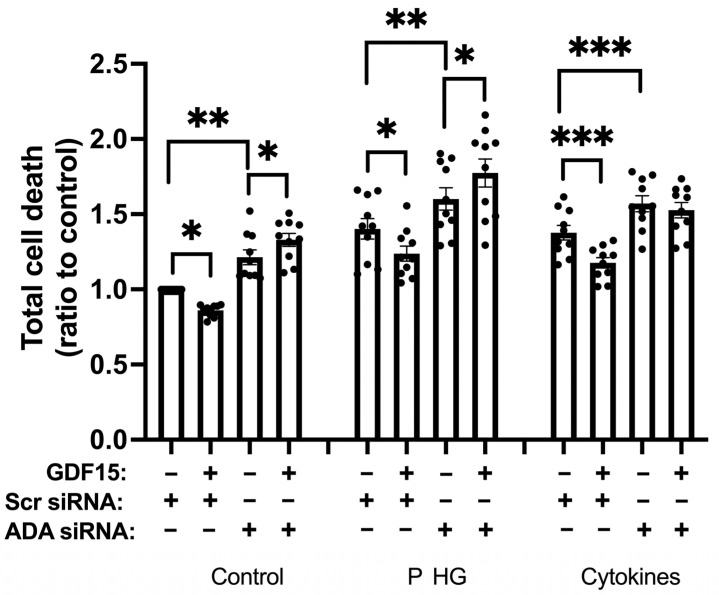
ADA activity mediates GDF15-induced protection against EndoC-betaH1 cell death. EndoC-betaH1 cells were lipofected with Scr or ADA siRNA one day prior to the start of the treatments. The cells were then preincubated with GDF15 (100 ng/mL) for 24 h followed by additional treatments with cytokines (20 ng/mL IL-1β + 20 ng/mL IFN-γ) or palmitate and high glucose (P HG, 1.5 mM palmitate + 22 mM glucose) for another 24 h. Cells were then labeled with Annexin V + PI and, subsequently, analyzed using a flow cytometer. The results are the ratios to scr siRNA control and are presented as the means ± SEM of 10 independent experiments; *, **, and *** denote *p* < 0.05, *p* < 0.01, and *p* < 0.001, respectively.

To assess whether the protective effect of GDF15 against IL-1β + IFN-γ- or palmitate + high-glucose-induced EndoC-betaH1 cell death requires ADA activity, cells were lipofected with control siRNA (scr) or siRNA targeted against ADA, and treated with GDF15, cytokines, and palmitate + high glucose as described in Figure 7. Cells were then labeled with PI and Annexin V and, subsequently, analyzed by flow cytometry. We observed that without ADA knockdown (scr siRNA), GDF15 protected against cell death under control, cytokine, and palmitate + high-glucose conditions (Figure 8). With ADA knockdown, however, GDF15 protection was no longer observed, under any condition. Instead, cell death in the presence of GDF15 increased under the control and palmitate + high-glucose conditions (Figure 8). These findings suggest that GDF15 counteracts beta-cell death using a mechanism facilitating increased ADA activity.

## 3. Discussion

We presently report that the addition of exogenous mature GDF15 protects partially against inflammatory and metabolic-stress-induced beta-cell dysfunction and death. The endogenous production of GDF15 in EndoC-betaH1 cells was low and the predominant intracellular form was pro-GDF15, which may not exhibit full GDF15 activity [27]. Thus, beta cells may rely, under basal conditions, on paracrine delivery of mature GDF15, rather than autocrine signaling. The beneficial effects of GDF15 were not immediate, as acute or short-term GDF15 exposures did not promote beta-cell protection (results not shown). Instead, a 24 h GDF15 preincubation period was routinely required for improved beta-cell function, suggesting that GDF15 acts via slow and complex transcriptional effects. That GDF15 promotes protection of human beta cells fits well with previous studies reporting beneficial effects in mouse beta-cell lines, mouse islets, and in in vivo mouse studies [18,19,20,21,22]. However, there seem to exist some differences between human and mouse beta cells, as glucolipotoxic conditions increased GDF15 expression in mouse [22] but not in human beta cells, as observed in the present study. According to RNA-sequencing data, human islets isolated from T2D and impaired glucose tolerance individuals do not express more GDF15 than control islets (https://mae.crc.med.lu.se/IsletGeneView/ (accessed on 15 December 2023)). In addition, not only species differences may explain this discrepancy, but it is also possible that different experimental protocols resulted in the variable pattern of GDF15 expression.

Imatinib but not metformin promoted increased pro-GDF15 levels in EndoC-betaH1 cells. We did not observe an elevated level of intracellular mature GDF15 in response to imatinib, but it is possible that the mature GDF15 is rapidly released from the cells so that an increased production of mature GDF15 is not easily detected. It is well known that imatinib protects beta cells against stress-induced dysfunction and death [25,26,34,35], and that this effect requires 6–24 h long preincubation periods. Therefore, it is tempting to propose that imatinib, by augmenting autocrine GDF15 signaling in beta cells, increases resilience to inflammatory or metabolic stress. It has been reported that AMPK activation promotes increased GDF15 production in hepatocytes and myotubes [15], which aligns well with our recent observation that imatinib, via mitochondrial inhibition, activates AMPK in beta cells [26]. We did not assess whether metformin, under the conditions used in the present study, promoted AMPK activation. However, as metformin stimulated AMPK activity in beta cells in a previous study [30], it may be that other signaling events, in addition to AMPK activation alone, are required for GDF15 production in beta cells, and that these are not sufficiently evoked by metformin.

In our hands, imatinib not only increased GDF15 production, but it also reduced the expression of thioredoxin-interacting protein (TXNIP) [26], a pro-oxidative protein that is induced by metabolic stress and that causes cellular dysfunction and death [36]. Interestingly, this resembles the situation in SH-SY5Y neurons overexpressing mitochondrial ferritin. In such cells, which are protected against stress, GDF15 is upregulated and TXNIP downregulated [37]. It is also noteworthy that metformin increases GDF15 and decreases TXNIP in cancer cells, an effect which has been suggested to contribute to metformin’s anticancer effects [38]. Clearly, the mechanisms by which imatinib and metformin, at least in some cases, regulate GDF15 and TXNIP in opposite directions, thereby achieving cellular protection, require further investigation.

It is well known that GDF15 is induced in response to mitochondrial, inflammatory, or metabolic stress [3], but, to our knowledge, the effects of GDF15 on mitochondrial respiration have not been previously studied. In human islets, GDF15 stimulated ATP-coupled respiration under basal conditions and the ECAR in cytokine-treated cells. Improvements in mitochondrial respiration and glycolytic flow rates may participate in GDF15-mediated beta-cell protection, as this will counteract cytokine-induced loss of ATP [28]. On the other hand, the metabolic improvements, promoted by GDF15 during cytokine stress, might instead reflect improved viability, and could, therefore, be a secondary phenotype rather than a direct cause of GDF15-induced protection.

On the one hand, low extracellular concentrations of adenosine have been reported to increase beta-cell insulin release and proliferation via adenosine receptor activation [39]. On the other hand, high intracellular adenosine concentrations are known to promote cell death in different cells with decreased capacity for adenosine disposal (ADA-deficiency), such as immune cells in ADA-severe combined immune deficiency, neuronal cells, endothelial cells, epithelial cells, and cancer cells [40,41,42,43,44,45]. We have observed that high adenosine levels are toxic to beta cells [33], suggesting that the inherent capacity of beta cells to handle buildup of intracellular adenosine is low. Therefore, by analyzing intracellular levels of inosine, the ADA-catalyzed deamination production of adenosine, in stressed beta cells, we could observe that GDF15 facilitated adenosine disposal. Furthermore, as siRNA-mediated downregulation of ADA resulted in both lowered inosine levels and loss of protection against stress-mediated cell death, it is possible that GDF15 acted as a beta-cell protection factor, at least in part, via ADA activation. Interestingly, cell death was, in some cases, higher in the GDF15-exposed cells with a simultaneous knockdown of ADA. The reason for this is not clear, but GDF15 might alter beta-cell metabolism so that adenosine production is accentuated, as suggested by the increased inosine levels, and when ADA fails in disposing this buildup, protection against cell death is turned into the opposite. Clearly, a better understanding of how GDF15 controls adenosine buildup and ADA activity is highly warranted.

A limitation of this study is that we only analyzed intracellular GDF15 and not released GDF15. Thus, it may be that imatinib and metformin evoked other effects on released GDF15 than those observed when analyzing intracellular GDF15. We also neither verified that AMPK mediates imatinib-induced GDF15 production nor studied how metformin and imatinib affect insulin secretion and content, viability, and ADA activity in beta cells exposed to GDF15. Furthermore, we did not analyze the effects of chronic GDF15 exposure, and it is possible, as GDF15 seems to promote pleiotropic effects, that long-term effects turn from beneficial to adverse. Indeed, it has been observed that GDF15 increases during aging and that this might accelerate different pathological processes [46].

## 4. Materials and Methods

### 4.1. Human Pancreatic Islet Culture

Isolated human pancreatic islets were kindly provided by Nordic Network for Clinical Islet Transplantation (Uppsala University Hospital, Uppsala, Sweden). Ethical permission to use human islet was reviewed and approved by the local ethics committee (Regionala etikprövningsnämnden, Uppsala, Sweden, Ups 03-515). After isolation, the islets were cultured free-floating in Sterilin dishes in CMRL 1066 medium (ICN Biomedicals, Costa Mesa, CA, USA) containing 5.6 mM glucose, 10% (*v*/*v*) fetal calf serum, antibiotics, and 2 mM L-glutamine at 37 °C in humidified air containing 5% CO_2_ for 1–5 days.

### 4.2. Human EndoC-betaH1 Cell Culture

Human EndoC-betaH1 cells were maintained in GeltrexTM (Gibco Billings, MT, USA)-coated plates in DMEM/Ham’s F12 (Gibco) (50%/50%, *vol/vol*, 5.5 mM glucose) medium supplemented with 2% fatty acid free bovine serum albumin (BSA) fraction V, 2 mM L-glutamine, MycoZap Plus PR, as previously described [47]. Cells were cultured in a humidified atmosphere at 37 °C with 5% CO_2_.

### 4.3. Immunocytochemistry (ICC)

Human islets were trypsinized so that a cell suspension was obtained, and then transferred to a clearly defined area of poly-lysine glass slides using a Cytospin 4 centrifuge (Thermo Scientific, Waltham, MA, USA). Thereafter cells were fixed with 4% (*w*/*v*) paraformaldehyde for 5–10 min at room temperature, blocked for 30 min using 2% BSA, permeabilized with 0.1% Tris-NaCl for 10 min, and stained overnight with the following primary antibodies: rabbit anti-GDF15 (Abcam, 1:250), guinea pig anti-insulin (Fitzgerald, Sudbury, MA, USA, 1:250), and mouse anti-glucagon (Thermo Fischer Scientific, 1:1000). All slides were washed with phosphate-buffered saline (PBS) and, prior to counterstaining, with diluted secondary antibodies: ALEXA Fluor 594 donkey anti-rabbit (Jackson, bar Harbor, ME, USA, 1:250), ALEXA Fluor 488 donkey anti-guinea pig (Jackson, 1:250), and ALEXA Fluor 594 goat anti-mouse (Life technologies, Carlsbad, CA, USA, 1:250) for 1 h at room temperature. Cells were washed with PBS and then mounted with Biotium’s (Freemont, CA, USA) EverBrite™ mounting medium containing 4’,6-diamidino-2-phenylindole (DAPI) for nuclei staining. Images were taken and analyzed with a Nikon Eclipse C1/TE2000-U microscopy (Nikon, Konan, Tokyo, Japan).

### 4.4. Immunoblot Analysis

EndoC-betaH1 cells were preincubated with 10 µM imatinib or 0.5 mM metformin for 6 h. This was followed by treatment with or without the combination of palmitate (1.5 mM) and glucose (22 mM) for an additional 24 h, still in the presence of imatinib or metformin. After the exposure period, total protein lysates were prepared using SDS sample buffer, at pH 6.8, containing Halt Phosphatase Inhibitor Cocktail (Thermo Fisher Scientific, Waltham, MA, USA). Samples were boiled for 5 min and separated on SDS-PAGE (4–20%, Bio-Rad, Hercules, CA, USA). Proteins were electrophoretically transferred to a Hybond PVDF membrane (GE Healthcare, Chicago, IL, USA) followed by blocking with 1% (*w*/*v*) BSA for 1 h. The membrane was then incubated with rabbit anti-GDF15 (1:1500, Abcam), mouse anti-beta-actin (1:1500, Santa Cruz, Santa Cruz, CA, USA), and mouse anti-alpha-tubulin (1:1500, Santa Cruz) antibodies overnight at 4 °C. Fluorescent anti-mouse (LI-COR Biosciences, Lincoln, NE, USA, IRDye) and anti-rabbit (LI-COR, IRDye) secondary antibodies, diluted to 1:15,000, were applied for 1 h at room temperature. Immunodetection was visualized and quantified using the LI-COR Odyssey Fc system (LI-COR).

### 4.5. Evaluation of Cell Viability

EndoC-betaH1 cells were preincubated with 0, 10, 100, or 1000 ng/mL of recombinant human GDF15 (ACROBiosystems, Newark, DE, USA) for 24 h before being cultured with 20 ng/mL IL-1β (Peprotech, Rocky Hill, NJ, USA) + 20 ng/mL IFN-γ (Peprotech, Rocky Hill, NJ, USA) or 1.5 mM palmitate + 22 mM glucose in the presence or absence of GDF15 for next 24 h. The concentrations of glucose and palmitate used are clinically relevant and have previously been observed to cause beta-cell dysfunction and death [47]. For human islets, cells were exposed to GDF15 and cytokines, as given above, and then cells were vital stained by incubation in medium containing 10 µg/mL bisbenzimide (Hoechst 33342, Sigma-Aldrich, Saint Louis, MO, USA) and 10 µg/mL propidium iodide (PI, Sigma-Aldrich) for 15 min. Red fluorescence (PI) and blue fluorescence (bisbenzimide) were examined using fluorescence microscopy (Nikon Eclipse C1/TE2000-U microscopy). The ratio of PI over bisbenzimide was calculated as a relative measure of cell death. For EndoC-betaH1 cell analysis, free-floating cells and attached cells were collected, combined, and stained with Annexin V-FITC/PI assay (Invitrogen, Carlsbad, CA, USA, cat# V13242A) according to the manufacturer’s protocol [34]. Fluorescent signals were evaluated using a BD Accuri C6 plus (BD Biosciences, Franklin Lakes, NJ, USA) flow cytometer. The percentage of apoptotic and necrotic cells was analyzed with BD Accuri C6 plus software (version 1.0.23.1) and combined to obtain total cell death rates (BD Biosciences).

### 4.6. Cell Proliferation

EndoC-betaH1 cells were seeded in 48-well culture plates (3 × 10^4^ cells/well). One day later (day 0), the cells were treated without or with 10 ng/mL or 100 ng/mL GDF15. The number of cells in each well was then determined using an Accuri C6 plus flow cytometer (BD Biosciences, Franklin Lakes, NJ, USA) from day 0 to day 4.

### 4.7. Glucose-Stimulated Insulin Secretion (GSIS)

EndoC-betaH1 cells were preincubated with GDF15 at the concentrations of 0, 10, or 1000 ng/mL for 24 h. The cells were then either left untreated or treated with a mixture of cytokines (20 ng/mL IL-1β + 20 ng/mL IFN-γ) or 1.5 mM palmitate + 22 mM glucose in the presence or absence of GDF15 for the next 24 h. Krebs-Ringer bicarbonate HEPES buffer (KRBH) containing 0.5% BSA and 1.7 mM glucose was added after the treatment medium was removed, and the cells were preincubated for 30 min at 37 °C. Thereafter, the cells were incubated with 16.7 mM glucose in KRBH + 0.5% BSA buffer at 37 °C for another 60 min. Insulin was then analyzed in cell supernatants collected from 16.7 mM glucose exposed cells. In order to measure the insulin content, cells were harvested and homogenized by sonication in distilled water. Samples were mixed (1:2) with 95% acid ethanol and kept for 2 h at −20 °C prior to supernatant separation. Insulin levels were then evaluated using the insulin ELISA assay kit (Mercodia, Uppsala, Sweden), and the Bradford protein assay was used to determine the total protein content.

### 4.8. Oxygen Consumption and Extracellular Acidification Rates

The oxygen consumption rates (OCRs) and extracellular acidification rates (ECARs) of isolated human islets were determined using the Extracellular Flux Analyzer xFe96 (Seahorse Bioscience, Billerica, MA, USA). Human islets were precultured without or with GDF15 (10 ng/mL or 100 ng/mL) for 24 h and then with an additional mixture of cytokines (IL-1β 20 ng/mL + IFN-γ 20 ng/mL) for 48 h. After the treatment, 8–10 human islets (6 replicates for each treatment group) were placed into each well of the xFe 96 cell culture microplate precoated with 3% ECM gel (Sigma-Aldrich) and 5 μg/mL fibronectin (Sigma-Aldrich). Islets were preincubated for 2 h at 37 °C in Seahorse Biosciences’ XF assay medium supplemented with 5.5 mM glucose. Following 20 min of basal recording, the cells were sequentially injected with oligomycin (Sigma Aldrich), carbonyl cyanide-p-trifluoromethoxyphenyl-hydrazone (FCCP) (Sigma Aldrich), and a combination of rotenone (Sigma Aldrich) and antimycin A (Sigma Aldrich) at intervals of 20 to 30 min. Each substance was introduced at the concentration of 5 µM. Measurements of the ATP-coupled OCR, maximum respiratory capacity, and ECAR were used to assess mitochondrial function. The data were normalized to protein contents determined using the DC protein assay (Bio-Rad).

### 4.9. siRNA-Mediated Silencing of Adenosine Deaminase (ADA)

An estimated 5 × 10^4^ EndoC-betaH1 cells were seeded onto 48-well plates, and cells were kept in culture for 48 h. The culture medium was removed and replaced by 250 µL of Opti-MEM (Gibco). Transfection was performed by using ADA siRNA (ON-TARGETplus SMARTpool human ADA, Dharmacon, Lafayette, CO, USA) or a Mission negative control siRNA (Sigma Aldrich). siRNA was diluted in 25 µL of Opti-MEM before combining with 0.5 µL of Lipofectamine™ RNAiMAX (Invitrogen, Carlsbad, CA, USA) diluted in 25 µL Opti-MEM. The entire combination was then diluted in total 300 µL of Opti-MEM to obtain the final concentration of 40 nM. Cells were incubated with the mixture for 6 h at 37 °C, after which the standard culture medium was added in place of the transfection medium. 

### 4.10. Inosine Detection

After applying ADA siRNA as previously mentioned, EndoC-betaH1 cells were preincubated without or with 10 ng/mL or 100 ng/mL of GDF15 for 24 h. Following that, cells were treated with cytokines or palmitate and high glucose over the next 24 h either with or without GDF15. After the treatment, medium was removed and replaced with KRBH buffer containing 5.5 mM glucose. Exogenous adenosine was added to the buffer at a concentration of 40 μM, and cells were incubated for 1 h at 37 °C. The supernatants were then collected for extracellular inosine determination. For intracellular inosine assessment, cells were deproteinized in iced-cold 0.4 M perchloric acid and centrifuged at 13,000× *g* for 10 min at 4 °C to remove insoluble materials. Supernatants were collected and neutralized using 4 M potassium carbonate solution. Neutralized supernatants were centrifuged at 13,000× *g* for 10 min at 4 °C and used for inosine analysis. Following the manufacturer’s instructions, the inosine assay kit (Sigma-Aldrich, catalog # MAK100) was used to measure the concentrations of inosine and hypoxanthine. The pellet from the perchloric acid homogenization step was dissolved by adding 0.5 M sodium hydroxide solution and used for Bradford protein analysis to determine the total protein concentration.

### 4.11. Data Analysis

Results are presented as the means ± SEM. Statistical analysis was performed with GraphPad Prism Version 6.0 (GraphPad software, San Diego, CA, USA). Statistical significance among several groups was evaluated using repeated measurement one- or two-way ANOVA followed by the Holm–Sidak multiple comparison post hoc test. *p* < 0.05 was considered statistically significant.

## 5. Conclusions

In conclusion, our findings suggest that imatinib-induced upregulation of pro-GDF15, or a 2-day exposure to exogenous mature GDF15, reduced beta-cell dysfunction and death caused by pro-inflammatory cytokines or palmitate + high glucose. This effect of GDF15 may have been mediated by increased disposal of intracellular adenosine.

## Data Availability

The data are available upon request.

## Supplementary Materials

The following supporting information can be downloaded at: https://www.mdpi.com/article/10.3390/ijms25020801/s1.

## Abbreviations

ADA	Adenosine deaminase
AMPK	AMP-dependent kinase
BSA	Bovine serum albumin
ECAR	Extracellular acidification rate
DAPI	4’,6-Diamidino-2-phenylindole
FCCP	Carbonyl cyanide-p-trifluoromethoxyphenylhydrazone
FPKM	Fragments per kilobase of exon model per million reads mapped
GDF15	Growth differentiation factor 15
GFRAL	Glial cell-derived neurotrophic factor family receptor alpha-like
IFN-γ	Interferon-γ
IL-1β	Interleukin 1β
KRBH	Krebs-Ringer bicarbonate buffer
OCR	Oxygen consumption rate
PI	Propidium iodide
RET	Rearranged during transfection
T1D	Type 1 diabetes
T2D	Type 2 diabetes
TXNIP	Thioredoxin-interacting protein
