# Supplementary material for: GDF15 Protects Insulin-Producing Beta Cells against Pro-Inflammatory Cytokines and Metabolic Stress via Increased Deamination of Intracellular Adenosine
Source: Int J Mol Sci. 2024 Jan 8;25(2):801. doi: 10.3390/ijms25020801 (PMC10815691; doi:10.3390/ijms25020801)
Supplement: Supplementary file 1 [file ijms-25-00801-s001.zip › ijms-2781862-supplementary.pdf]

Supplementary Figures

Supplementary Figure S1

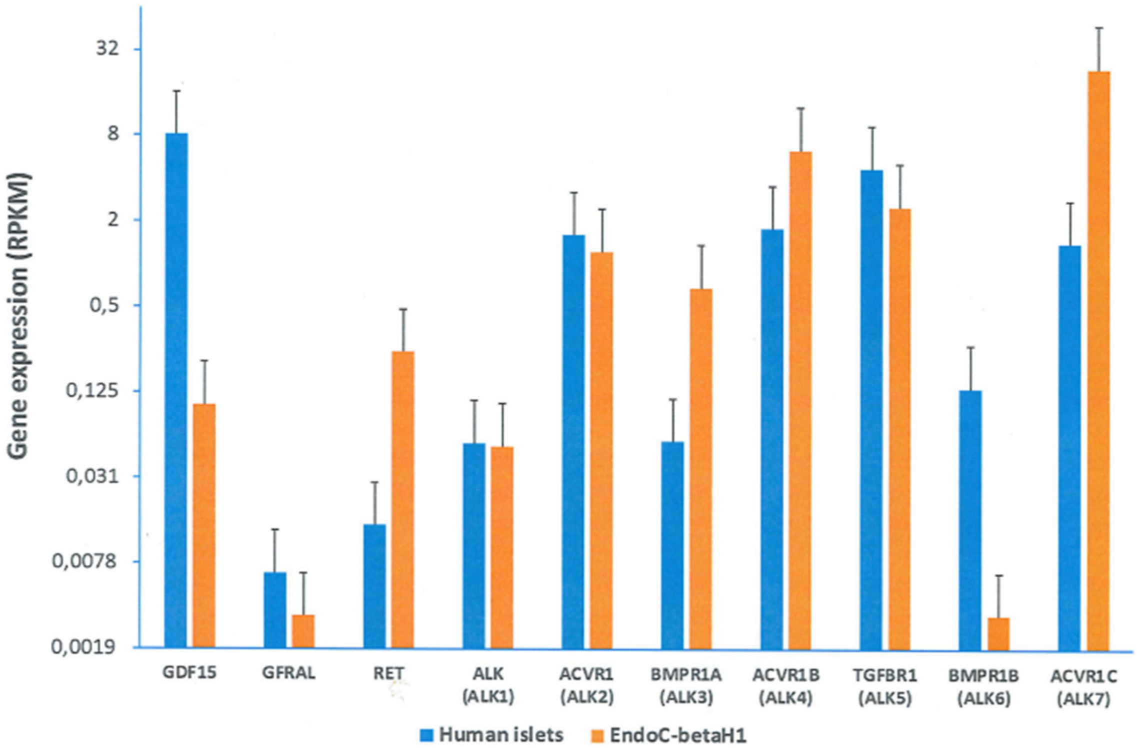

Figure S1. Expression of GDF15 and putative GDF15 receptors in human islets and EndoC-betaH1 cells. Gene expression levels were obtained by RNA-seq and data have been compiled from a previous publication [28]. Results are shown as mean RPKM ± SEM (n=3).

Supplementary Figure S2

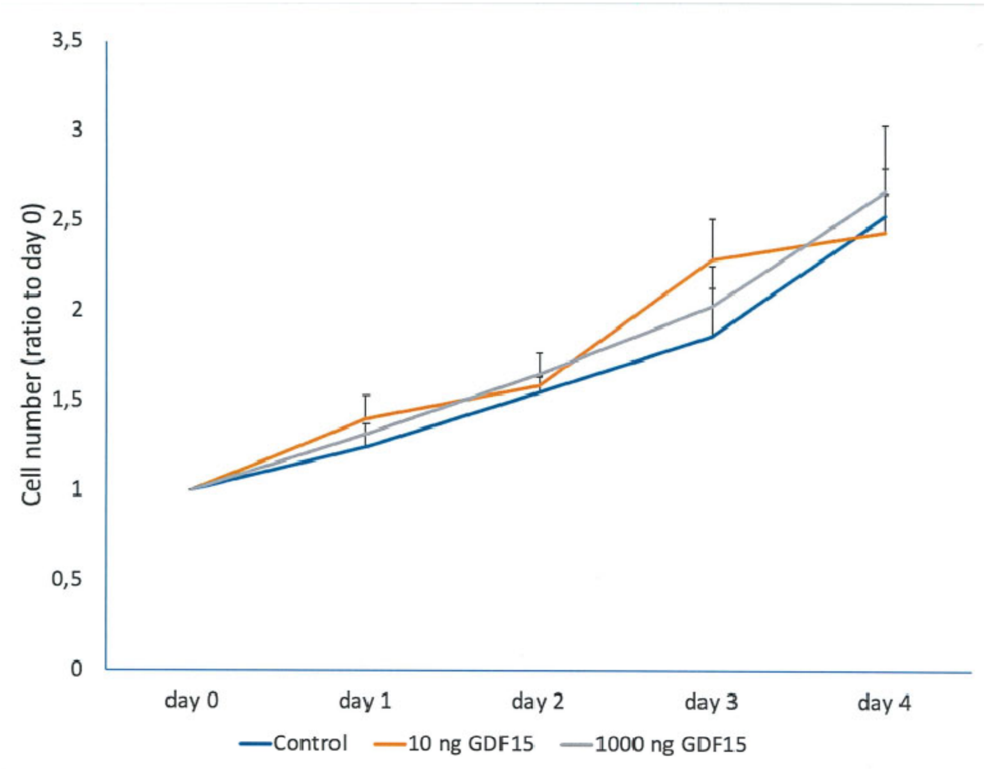

Figure S2. EndoC-betaH1 cell proliferation is not affected by GDF15. Cells were incubated without or with 10 ng/ml or 1000 ng/ml of GDF15 throughout the 4 days. Total cell numbers, at day 0, 1, 2, 3, and 4, were obtained by flow cytometry analysis. The ratios of day 1-4 to that on day 0 was analyzed and plotted. Results are mean ± SEM from 6 independent experiments.
